# Development and Validation of an Animal Model of Prostate Inflammation-Induced Chronic Pelvic Pain: Evaluating from Inflammation of the Prostate to Pain Behavioral Modifications

**DOI:** 10.1371/journal.pone.0096824

**Published:** 2014-05-13

**Authors:** Feng Zeng, Hequn Chen, Jinrui Yang, Long Wang, Yu Cui, Xiao Guan, Zhao Wang, Jiping Niu, Xiongbing Zu, Lin Qi, Xiangyang Zhang, Zhengyan Tang, Longfei Liu

**Affiliations:** 1 Department of Urology, Xiangya Hospital, Central South University, Changsha, Hunan, China; 2 Department of Urology, The Second Xiangya Hospital, Central South University, Changsha, Hunan, China; Northwestern University, United States of America

## Abstract

**Background:**

Chronic prostatitis/Chronic pelvic pain syndrome (CP/CPPS) is the most common type of prostatitis. Due to the lack of a suitable animal model partly, the pathogenesis for this condition is obscure. In the current study we developed and validated an animal model for nonbacterial prostatitis and prostate inflammation-induced chronic pelvic pain in rats with the use of intraprostatic injection of λ-carrageenan.

**Methods:**

Male Sprague-Dawley rats weighing 250–350 g were used for the experiments. After intraprostatic injection of 3% λ-carrageenan, at different time points(after 24 h, 7d, 14d and 30d of injection), radiant heat and von Frey filaments were applied to the scrotum of rats to measure the heat and mechanical thresholds respectively. Then the prostate was removed for histology, and cyclooxygenase (COX) 2 protein expression was determined by Western-blot. Evans blue(50 mg/kg) was also injected intravenously to assess for plasma protein extravasation at different time points after injection of λ-carrageenan.

**Results:**

Compared to control group, inflamed animals showed a significant reduction in mechanical threshold (mechanical allodynia) at 24 h and 7d(*p* = 0.022,0.046, respectively), and a significant reduction in heat threshold (thermal hyperalgesia) at 24 h, 7d and 14d(*p* = 0.014, 0.018, 0.002, respectively) in the scrotal skin. Significant increase of inflammatory cell accumulation,COX2 expression and Evans blue extravasation were observed at 24 h, 7d and 14d after injection.

**Conclusions:**

Intraprostatic λ-carrageenan injection induced neurogenic prostatitis and prostate inflammation pain, which lasted at least 2 weeks. The current model is expected to be a valuable preclinical tool to study the neurobiological mechanisms of male chronic pelvic pain.

## Introduction

Chronic prostatitis/Chronic pelvic pain syndrome (CP/CPPS) is the most common type of prostatitis, which accounted for approximately 90% of this prevailing disease. Its current definition by National Institutes of Health (NIH) is genitourinary pain with or without voiding symptoms in the absence of uropathogenic bacteria or other identifiable causes such as malignancy[1]. Chronic pain is the most common and disturbing symptom of CP/CPPS, and can arise in the pelvic area, penis and testicles, or on urination and ejaculation[2]–[4]. In the NIH-Chronic Prostatitis Symptom Index (NIH-CPSI), chronic pain is considered as a major symptom of chronic prostatitis, along with urinary symptoms and reduction in quality of life[2], bringing a huge economic and psychological burden to the patients and society. However, the etiology and mechanism of pain development is unclear.

A suitable preclinical animal model of prostatitis-induced pain or prostatodynia can help us to understand this clinical issue sufficiently. Over the last three decades, investigators have developed several animal models including immune-induced prostatitis models, hormone-associated prostatitis models and other miscellaneous prostatitis models, in hope to creating a novel reliable animal model of prostatitis to uncover the etiology and pathogenesis of CP/CPPS[5]. However, the vast majority of the animal models just focused on the inflammation of the prostate, without characterizing the pain behavior caused by inflammatory prostatitis.

Carrageenan is a polysaccharide which is very commonly used to induce inflammation and subsequent pain in various inflammatory pain models[6]. In the present study, we modeled prostatic inflammation and pain by intraprostatic λ-carrageenan injection in the rat. We determined the extent of inflammatory changes by measuring COX2 expression, plasma extravasation, and accumulation of inflammatory cells in the prostate, and assessed the behavioral pain thresholds of carrageenan-treated rats. Meanwhile, we extend the observation time up to 1 month to assess the duration of this model.

## Materials and Methods

### 1. Experimental animals

Adult male Sprague-Dawley rats (250–350 g body weight, n = 80) were used for the experiments. Animals were housed in groups of two or three in plastic cages with soft bedding and free access to food and water under a 12/12 hour reversed light-dark cycle (dark cycle: 8:00 a.m.-8:00 p.m.). All animals were acclimated for 1 week before any experimental procedures.

All experimental protocols were approved by the Animal Care and Use Committee of the Xiangya Hospital, Central South University. All surgery was performed under isoflurane anesthesia, and all efforts were made to minimize suffering.

### 2. Carrageenan injection

All the SD rats were randomly divided into two groups: model group and control group. For injection of carrageenan, rats were anesthetized with isoflurane (5% for induction and 3% for maintenance) and were fixed in a supine position. Then, the lower abdomen above the penis of rats was shaved and the skin in this area sterilized using 3 applications of 10% povidone-iodine solution. A small midline incision was made in the sterile area, then the bladder and the prostate carefully exposed. With a 30-gauge needle, 50 ul sterile suspension of 3% carrageenan (Sigma, MO, USA) was injected into both right and left ventral lobes of the prostate gland(model group, n = 40). For the control group, commensurable sterile normal saline was injected. After the injection, a 2% lidocaine solution was applied to the wound, and then the wound was closed in layers.

### 3. Behavioral testing for assessment of pain thresholds

At different time points(before and after 24 hours, 7 days, 14 days and 30 days of injection, n = 5), thresholds to heat stimulus and mechanical stimulus were measured. Because as Radhakrishnan[6] reported, model animals showed a significant reduction in mechanical threshold and heat threshold only in the scrotal skin, so in our research we did not test the pain threshold in the other areas including the ventral tail root and the area between the penis and the scrotum. All the measurement of pain thresholds were completed by an independent investigator (Y Cui) unaware of the animal grouping.

For the test of thresholds to heat stimulus, the rats in both groups were kept in Plexiglas cubicles on the glass platform of a plantar analgesia testing equipment (37370 Plantar Test, IUgo Basile,Italy) for 30 minutes to acclimatize. Radiant heat from a light source was shone on the skin of scrotal. The time duration from the beginning of test to the escape from the heat stimulus was noted as threshold to heat stimulus. Five measurements were applied in one rat and 5 minutes of resting time was provided between each application, and an average of 5 time readings was taken as the final data.

After 1 hour of resting time, threshold to mechanical stimulus of rats,which were placed in a wire mesh platform for 30 minutes to acclimatize,was tested using a series of von Frey filaments from 0.4 to 26 g(IITC Life Science, USA) applied one by one to the scrotal skin. The bending force of the filament to which the animal responded was taken as the baseline threshold to mechanical stimulus. Five measurements were applied in one rat and 5 minutes of resting time was provided between each application, and an average of 5 time readings was taken as the final data.

### 4. Histological analysis

At different time points(after 24 hours, 7 days, 14 days and 30 days of injection), after behavioral testing, a portion of rats(n = 5) were sacrificed and the prostate was harvested. For histological analysis, one part of the prostate was fixed in buffered 10% formaldehyde for 24 h, embedded in paraffin, cut with a microtome, and stained with hematoxylin-eosin. Under a low-power microscopy field(×400), each slide was evaluated randomly in 4 different areas containing inflammatory cells by a independent investigators (JP Niu) unaware of the the animal grouping. The rest of prostate was frozen in liquid nitrogen and stored until use for analysis of protein expression.

### 5. Cyclooxygenase-2 (COX2) expression evaluation

The COX2 expressions in prostate of both groups were determined by western blot analysis. First of all, the samples were mashed and homogenized l ysed in lysis buffer containing phenylmethyl sulfonyl flouride. The protein was extracted and the amount of total protein was measured with the Bradford protein assay method. Then, twenty ul protein extraction solution was resolved into sodium dodecylsulfate- polyacrylamide gel electrophoresis and transferred to PVDF Membrane. The membrane was immunoblotted with the use of a 1∶1000 dilution of COX2 goat polyclonal antibody (Santa Cruz Biotechnology, Inc), with incubation at 4°C overnight. Horseradish peroxidase-linked antigoat immuglobulin G was used as the secondary antibody, with incubating 2 h at room temperature. LabWorks Image Acquisition and Analysis software was used for quantitative analysis.

### 6. Plasma protein extravasation evaluation

At different time points(after 24 h, 7d, 14d and 30d of injection), some animals(n = 5) were used to evaluate the plasma protein extravasation after intraprostatic injection of λ-carrageenan. Evans blue (50 mg/kg; Sigma-Aldrich, Steinheim, Germany) was injected intravenously. After 30 minutes, an incision was made on the right atrial appendage, and a perfusion with normal saline was applied through the aorta till the perfusion fluid became limpid. Rats were sacrificed and the prostate was removed, blotted on a wet filter paper, weighed, and stored in formamide (3 ml; Sigma-Aldrich, Steinheim, Germany) at room temperature for 72 h to extract Evans blue from the tissues. The amount of Evans blue was quantified by measuring the optical density of the extracted dye at a wavelength of 620 nm with the use of a spectrophotometer, and then expressed as µg/g of wet tissue weight.

### 7. Statistical analysis

Data were presented as means±standard deviation. Multivariate ANOVA followed by Tukey's test was used to compare all the indexes in model *vs* control animals at different time points. Data were analysed using SPSS software, version 11.0 (SPSS Inc., Chicago, IL, USA) for Windows. A P-value <0.05 was considered to be statistically significant.

## Results

### 1. Inflammatory cell accumulation

**Figure 1:**
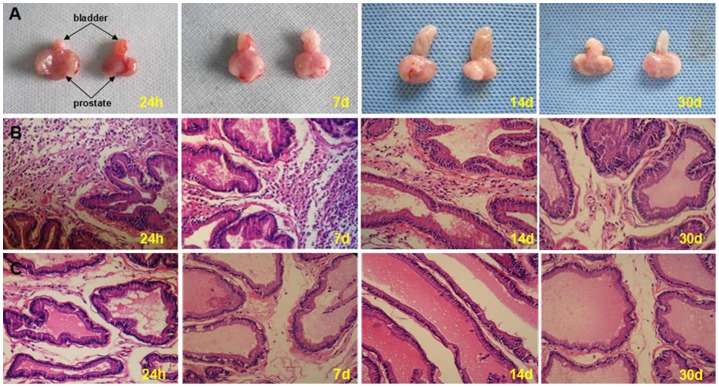
The histological detection after intraprostatic injection of carrageenan. A: Prostatic edema occurred at 24 h, 7d and 14d after intraprostatic injection of 3%λ-carrageenan, but at 30d after injection, no significant difference was observed in both groups(left: inflamed group; right: control group). B: Abundant inflammatory cells accumulated in the interstitial space at 24 h, 7d and 14d after intraprostatic injection of 3%λ-carrageenan. At 30d after injection, there was few inflammatory cell observed. C:There was no significant inflammatory cells accumulated in control.

Intraprostatic carrageenan injection induced prostatic edema (**Fig. 1A**) and increased inflammatory cell accumulation (**Table. 1**). A majority of inflammatory cells were neutrophils and presented to the interstitial space (**Fig. 1B**). These changes lasted at least 2 weeks, but less than 30 days, because the inflammatory cell count in model group was significantly higher than control group at 24 h, 7d and 14d after carrageenan injection.

**Table 1:** Effects of 3%λ-carrageenan on thermal hyperalgesia, mechanical allodynia, inflammatory cell count, COX2 expression and Evans blue extravasation at different time points(n = 5).

Parameters	Pre surgery	24 h	7d	14d	30d
Thermal hype -ralgesia(s)					
Carrageenan	26.42(1.81)	20.50(3.04)*	14.41(5.08)*	21.02(1.78)*	24.76(1.61)
Control	24.64(3.22)	25.48(1.82)	22.31(3.10)	28.44(3.20)	25.61(0.97)
Mechanical allodynia(g)					
Carrageenan	6.09(1.11)	3.90(1.32)*	2.84(1.63)*	6.10(1.60)	6.50(1.11)
Control	6.10(1.43)	8.14(3.06)	6.12(2.65)	5.90(1.48)	5.52(1.10)
Weights of the prostate(g)					
Carrageenan		0.2917(0.0335)*	0.2745(0.0187)*	0.2699(0.0277)*	0.2129(0.0242)
Control		0.2383(0.0344)	0.2086(0.0283)	0.2209(0.0274)	0.2087(0.0225)
Inflammatory cell count/HPF					
Carrageenan		275.4(27.0)*	311.8(77.4)*	152.4(56.5)*	35.2(12.2)
Control		35.4(14.5)	23.8(12.6)	33.0(13.9)	24.6(6.8)
COX2 expression					
Carrageenan		0.69(0.081)*	0.81(0.080)*	1.09(0.202)*	0.66(0.126)
Control		0.56(0.071)	0.55(0.075)	0.58(0.091)	0.58(0.630)
Evans blue (mg/g)					
Carrageenan		8.30(0.73)*	6.83(1.77)*	6.84(0.72)*	3.47(0.88)
Control		2.71(0.90)	2.69(1.21)	3.35(1.15)	2.84(0.74)

COX2 = cyclooxygenase 2.

Values in the parenthesis are standard deviation(SD).

*p<0.05 Compare to control group.

### 2. Mechanical allodynia and thermal hyperalgesia

Reductions in thresholds to mechanical or heat stimuli in the pelvic areas after inflammation are interpreted as heat hyperalgesia and mechanical allodynia respectively for the purpose of this study. A statistically significant reduction in average mechanical threshold of the rat scrotal skin was observed at 24 h and 7 d time points compared to the control animals (*p* = 0.022, 0.046, respectively) (**Table. 1**). These reduced mechanical thresholds gradually and steadily recovered to pretreatment levels by 14 d after carrageenan treatment. Also, the average heat threshold of rat scrotal skin was significantly decreased at 24 h, 7 d and 14 d after intraprostatic carrageenan injection (p = 0.014, 0.018, 0.002, respectively) (**Table. 1**).

### 3. Cyclooxygenase-2 expression

**Figure 2:**
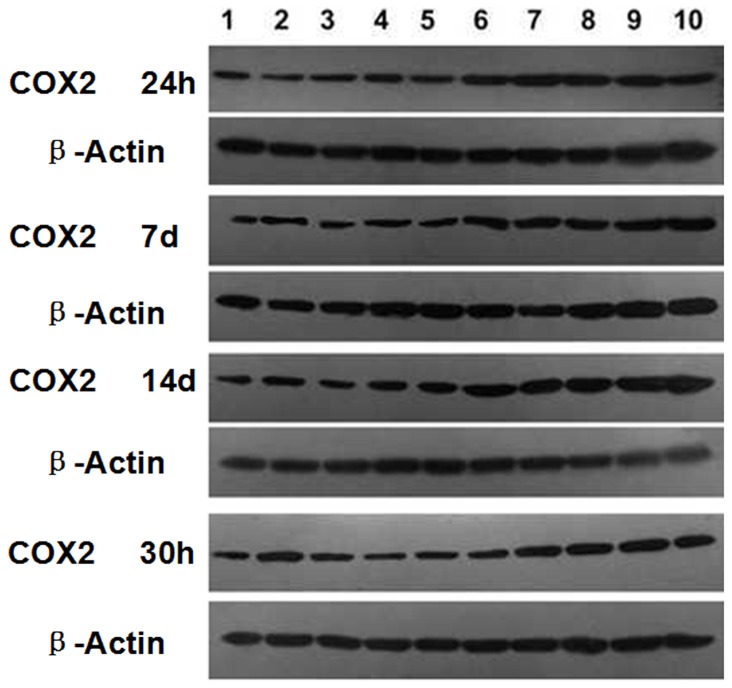
Comparison of cyclooxygenase-2 expression. 1–5: control group; 6–10: inflamed group. Intraprostatic injection of carrageenan induced significant increase of cyclooxygenase-2(COX2) expression at 24 h, 7d and 14d after injection.

As shown in **Fig. 2**, at 24 h, 7 d and 14 d after the injection of carrageenan, cyclooxygenase-2(COX2) expression in the rats prostate increased. Analysis of gray demonstrated this up regulation was statistically significant (**Table. 1**). Similar to the inflammatory cell accumulation, these changes lasted at least 2 weeks, but no more than 30 days, which implied inflammatory processes in prostate induced by carrageenan injection.

### 4. Plasma protein extravasation

**Figure 3:**
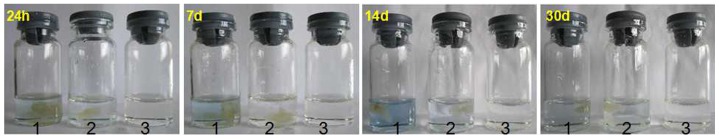
Comparison of Evans blue extravasation. 1: inflamed group; 2: control group; 3: blank control. Evans blue extravasation was significantly increased at 24 h, 7d and 14d after intraprostatic injection of carrageenan.

As shown in **Fig. 3**, after carrageenan injection, Evans blue extravasation was significantly increased in model group, which implied neurogenic plasma extravasation in the prostate. These increased Evans blue extravasation lasted at least 2 weeks and recovered to pretreatment levels at 30 d time point (**Table. 1**).

## Discussion

CP/CPPS is one of the most common entities encountered in urologic practice and represents an important international health problem[7]. The hallmark of CP/CPPS is the symptom complex of pelvic area pain and low urinary tract symptoms, along with reduction in quality of life (Qol)[2]–[4]. However, pain has more impact on Qol than urinary symptoms[3]. Despite the high prevalence and lack of optimal treatment, the mechanism of pain development in prostatitis is poorly understood. This is partly due to the lack of a suitable preclinical model of prostatitis-induced pain or prostatodynia[6]. Animals models mimic chronic pelvic pain will provide a unique opportunity to elucidate mechanisms involved in the molecular pathogenesis of CP/CPPS, which are complicated or even impossible to define in man.

There are several animal models of prostatitis developed in the past, including spontaneous, infectious models, hormone- and immune-induced, and some other models[5]. However, in most of these animal models, considerable attention has been focused on studying the basic histopathology and inflammatory mechanisms, not characterizing the pain behavior caused by inflammatory prostatitis. Recently, some researchers started to pay attention to the pain behavior in animal models. Quick et al[8] firstly described the methodology using von Frey fibers to quantify tactile allodynia in a murine model of bacterial prostatitis. Rudick et al[9] developed a mice model of experimental autoimmune prostatitis(EAP) by subcutaneous injection of prostate antigen, and in that model pelvic pain was detected 5 days after antigen instillation and was sustained beyond 30 days. Subsequently, Altuntas and colleagues[10] also reported elevated pelvic pain response, significantly higher micturition frequency and decreased urine output per void were observed in P25 99–118-immunized mice at 9 wk after immunization. These models maybe provide a useful tool for exploring the pathophysiology of CP/CPPS, but the cost of time and economy to modelling was a very important element to consider. Furthermore, the prostate tissue autoantigen involved in the induction of autoimmune prostatitis is not well characterized and NOD mice which was the most commonly used to develop antigen-induced prostatitis models frequently developed diabetes and other organ-specific autoimmune diseases that may interfere to specify study of prostate inflammation[5].

Chemical irritations such as formalin, capsaicin, dinitrobenzenesulfonic acid and ethanol has been used to establish animal models of chronic prostatitis/Chronic pelvic pain syndrome (CP/CPPS) by intraprostatic injection[11]–[14]. The disadvantages of these chemicals were that they could cause obvious tissue destruction and most of the induced-inflammation was inclined to a transient acute inflammation. Lang *et al* [12] reported ethanol plus dinitrobenzenesulfonic acid can result in significant mucosal injury. And, in a study of the model of nonbacterial prostatitis, modifications in pain behavior such as closing of the eyes, hypolocomotion, and inflammatory changes(increase of inflammatory cell accumulation, COX2 expression, and plasma extravasation)were developed after intraprostatic capsaicin injection in rats, but completely recovered at 1 week[13]. They considered this animal model can not reproduce the chronic character of the nonbacterial prostatitis in men. In our study, an animal model for prostate inflammation pain in rats was developed with the use of intraprostatic injection of carrageenan, and the reliability and validity of this model was tested by changes in pain behavior and the extent of inflammatory changes of the prostate.

Different from these chemicals, 3% λ-carrageenan can cause chronic reductions in threshold to heat stimulus and mechanical stimulus in rats without excessive tissue damage[15]. Radhakrishnan *et al* [6] first developed an animal model of inflammation-induced pelvic pain (NIH category III)with the use of intraprostatic injection of 3% λ-carrageenan in SD rats. They found inflamed animals produced by intraprostatic carrageenan injection showed a significant reduction in mechanical and heat threshold in the scrotal skin, and morphine (5 mg/kg. i. p.) can significantly increase both thresholds in the scrotal skin of model animals compared to normal saline, so they thought such nocifensive behavior as reduction of pain threshold caused by carrageenan was indeed indicative of pain. However, they did not evaluate about the inflammation in prostate and pain behavior was assessed just for 1 week. Recently, Chen *et al* [16] reported intraprostatic carrageenan injection can cause tactile allodynia in the scrotal base and the formation of inflammasome in prostate of model animals. They found the bearing threshold of withdrawal response in carrageenan treated rats was 80.0±11.0% of the initial pre-treatment threshold and chlorogenic acid treatment relieved the scrotal hypersensitivity. Our study also demonstrated that intraprostatic 3% λ-carrageenan injection induced significant reduction of mechanical and heat thresholds in the scrotal skin, and it was more important that the time of this change lasted(at least 2 weeks) in this animal model was longer than the others. And there was no tissue destruction observed in pathological examination of rats prostate.

The principal symptom of CP/CPPS is pain, which can affect the perineum, the anus, the bladder region, the testicles, the penis and the groin region[2]–[4]. Scholars has suggested that neurological factor was involved in the pain of prostatitis by the finding that chemical irritation of rat prostate and bladder causes c-fos expression at spinal cord levels L6 and S1, along with plasma extravasation in the skin at the identical L6 and S1 dermatomes[11]. Rudick also considered spinal and/or supraspinal contribution to chronic pain in their mice model of experimental autoimmune prostatitis(EAP) because they found the neuromodulator gabapentin could significantly remit the pelvic pain in EAP mice[9].Yang and colleagues[17] compared thermal algometry in men with CP/CPPS and asymptomatic controls and considered that central sensitization played a very important role in these patients. Also, In our research, plasma extravasation was increased in the prostate of carrageenan-treated rats, which suggested carrageenan can induce neurogenic inflammation as same as the other chemicals such as capsaicis[11], [13]. We also deemed this carrageenan-induced pain in our model rats was similar to the painful condition that puzzled CP/CPPS patients.

Other findings of this study are that intraprostatic 3% λ-carrageenan injection can induce inflammatory cell infiltration and COX2 expression increased in the rats prostate. Cyclooxygenase was a key enzyme of prostaglandin synthesis which was observed in 2 isoforms: COX1 and COX2[18]. Inflammatory processes facilitated the up-regulation of isoform COX-2. Then, COX-2 derived prostaglandins, particularly prostaglandin E2, which was the primary pathogenic factor of symptoms such as pain, fever and swelling, representing the classic triad of inflammation[19], [20]. Chen *et al* [16]found a close relationship between activation of inflammasome and pathophysiologic changes of rats treated by carrageenan. We considered this change may be a general character of all the chemical irritations because Chuang and his colleagues reported that an inflammatory reaction, including increases in inflammatory cell accumulation and COX2 expression were observed in prostate after intraprostatic capsaicin injection[13], [14].

One of the shortcomings in our study is that the interval of time point of observation after injection was too long to know accurately how long this carrageenan-induced inflammation lasted in the model rats, which need further research. Beyond that, it is common in clinic that CP/CPPS patients have significant symptoms but the inflammation of prostate is mild or absent[21]. But the present model show a correlation between prostatic pain and inflammation. We attribute this to the difference of prostate anatomy and physiology between human and rat, and also, we could not ignore this model is developed by the acute stimulation of carrageenan.

## Conclusions

Intraprostatic λ-carrageenan injection induced neurogenic prostatitis and prostate inflammation pain, which lasted at least 2 weeks. The current model is expected to be a valuable preclinical tool to study the neurobiological mechanisms of male chronic pelvic pain.
